# Isolation of TRPV1 independent mechanisms of spontaneous and asynchronous glutamate release at primary afferent to NTS synapses

**DOI:** 10.3389/fnins.2014.00006

**Published:** 2014-01-31

**Authors:** Axel J. Fenwick, Shaw-wen Wu, James H. Peters

**Affiliations:** Department of Integrative Physiology and Neuroscience, Washington State UniversityPullman, WA, USA

**Keywords:** vagus, solitary tract, vesicle release, synaptic, calcium, autonomic reflexes, TRPV3, TRPM3

## Abstract

Cranial visceral afferents contained within the solitary tract (ST) contact second-order neurons in the nucleus of the solitary tract (NTS) and release the excitatory amino acid glutamate via three distinct exocytosis pathways; synchronous, asynchronous, and spontaneous release. The presence of TRPV1 in the central terminals of a majority of ST afferents conveys activity-dependent asynchronous glutamate release and provides a temperature sensitive calcium conductance which largely determines the rate of spontaneous vesicle fusion. TRPV1 is present in unmyelinated C-fiber afferents and these facilitated forms of glutamate release may underlie the relative strength of C-fibers in activating autonomic reflex pathways. However, pharmacological blockade of TRPV1 signaling eliminates only ~50% of the asynchronous profile and attenuates the temperature sensitivity of spontaneous release indicating additional thermosensitive calcium influx pathways may exist which mediate these forms of vesicle release. In the present study we isolate the contribution of TRPV1 independent forms of glutamate release at ST-NTS synapses. We found ST afferent innervation at NTS neurons and synchronous vesicle release from TRPV1 KO mice was not different to control animals; however, only half of TRPV1 KO ST afferents completely lacked asynchronous glutamate release. Further, temperature driven spontaneous rates of vesicle release were not different from 33 to 37°C between control and TRPV1 KO afferents. These findings suggest additional temperature dependent mechanisms controlling asynchronous and thermosensitive spontaneous release at physiological temperatures, possibly mediated by additional thermosensitive TRP channels in primary afferent terminals.

## Introduction

Primary vagal afferent neurons provide a direct neural pathway through which the ongoing status of visceral organ systems (including the heart, lungs, and gastrointestinal tract) is conveyed to the brain (Loewy, 1990). Vagal afferents contact neurons within the nucleus of the solitary tract (NTS) to initiate homeostatic reflex pathways and inform the relevant forebrain projecting neurocircuitry on the ongoing peripheral status (Saper, 2002). The range of information types, time-frames, and relative physiological urgencies conveyed by vagal afferents is remarkable. As a result information transfer must be at one time reliable and precise while maintaining plasticity to match autonomic function to the physiological state. Vagal afferents release the fast excitatory neurotransmitter glutamate via three distinct pathways: synchronous, asynchronous, and spontaneous (Andresen et al., 2012b). Synchronous glutamate release is a tightly regulated process by which an action potential evokes a coordinated release of multiple glutamate vesicles into the synaptic cleft to ensure high-fidelity signaling between neurons. In contrast, asynchronous glutamate release is a loosely timed process by which an action potential evokes a release of glutamate that is delayed in response to the action potential. Equally significant is spontaneous glutamate release, by which neurotransmitter is released independent of action potential depolarization of the terminal. We predict that the interplay between multiple complementary vesicle release pathways allows for robust/precise information transfer which is also adaptable and plastic to changing physiological states.

Centrally, vagal afferent neurons merge with the facial (VII) and glossopharyngeal (IX) nerves to form the solitary tract (ST) bundle and innervate second-order NTS neurons (Loewy, 1990). ST-NTS synapses are a strong high probability of release (P_*r*_) synapse where action potential invasion of the central terminals leads to the coordinated synchronous release of multiple vesicles (Bailey et al., 2006b; Peters et al., 2008). Synchronous vesicle release provides a large excitatory drive from the afferent to the postsynaptic neuron and ensures high fidelity synaptic throughput (Bailey et al., 2006a). While synchronous release is large initially, during trains of action-potentials at physiological frequencies it rapidly falls off due to vesicle release outpacing vesicle mobilization to the membrane; a process called frequency dependent depression (FDD) (Chen et al., 1999; Doyle and Andresen, 2001). The presence of the transient receptor potential vanilloid type 1 (TRPV1) ion channel in the central terminals of most ST afferents conveys activity-dependent asynchronous glutamate release prolonging the period of action potential driven release (from milliseconds to seconds) and extending the postsynaptic excitatory period despite FDD of synchronous release (Peters et al., 2010). TRPV1 also provides a temperature sensitive calcium conductance which largely determines the rate of spontaneous vesicle fusion (Shoudai et al., 2010). Further, convergent vagal afferent innervation onto NTS neurons is segregated based on the presence or absence of TRPV1 and asynchronous vesicle release (Peters et al., 2011). Because TRPV1 is present in unmyelinated C-fiber afferents these facilitated forms of glutamate release may underlie the relative strength of C-fibers in activating autonomic reflex pathways (Peters et al., 2010; Andresen et al., 2012b).

The current experiments aim to further determine the contribution of TRPV1 in synaptic communication at ST-NTS synapses. As previously reported pharmacological blockade of TRPV1 signaling eliminates only ~50% of the asynchronous profile (Peters et al., 2010) and attenuates the temperature sensitivity of spontaneous release (Shoudai et al., 2010) indicating additional thermosensitive calcium influx pathways may exist to mediate these forms of vesicle release. In the present study we utilize mice genetically lacking the TRPV1 ion channel (TRPV1 KO) and characterize synchronous, asynchronous, and spontaneous vesicle release pathways as well as afferent innervation patterns independent of TRPV1. In addition we provide evidence that alternative thermo-TRP channels are functionally expressed in primary vagal afferent neurons.

## Methods

### Animals

Male Sprague Dawley rats (120–250 g, Simonsen Laboratories), male C57BL/6 mice (20–30 g), and male TRPV1^−/−^ knockout mice (20–30 g) (B6.129X1-*Trpv1*^*tm*1*Jul*^/J, Jackson Laboratories) were used under procedures approved by the IACUC at Washington State University. The TRPV1 gene product was confirmed as present (wild type, WT) or lacking (TRPV1^−/−^) via PCR amplification of genomic DNA and with RT-PCR on collected nodose ganglia. Animals were housed under 12 h light/12 h dark conditions and fed standard pellet chow *ad libitum*.

### Molecular biology

Wild-type and homozygous TRPV1 null mice were purchased from Jackson Laboratory and genotyped according to protocol for separated PCR. Genomic DNA was isolated from tail fragments using NaOH extraction. cDNA was obtained from RNA isolated from nodose ganglia mice. RNA (purified using the Qiagen RNEasy Kit and the QIACube robotic workstation) was converted into cDNA using the Ambion DNA-free Kit and QuantiTect Reverse Transcription Kit. Primers and cycling parameters used to detect WT TRPV1 fragments from genomic DNA and cDNA were obtained from the Jackson Laboratory website for genotyping using separated PCR: forward—CCT GCT CAA CAT GCT CAT TG; reverse—TCC TCA TGC ACT TCA GGA AA. Expected sizes for Wild-type TRPV1 fragments are 984 bp for genomic DNA and 134 bp for cDNA.

### Horizontal brainstem slice preparation

Experiments utilizing brainstem slices were performed on WT and TRPV1^−/−^ mice anesthetized with isoflurane as previously described (Doyle et al., 2004). The medulla was removed and placed in ice-cold artificial cerebral spinal fluid (aCSF) containing (mM): 125 NaCl, 3 KCl, 1.2 KH_2_PO_4_, 1.2 MgSO_4_, 25 NaHCO_3_, 10 dextrose, and 2 CaCl_2_, bubbled with 95% O_2_–5% CO_2_. Once chilled, the tissue block was trimmed to remove the remaining cerebellum and a wedge was taken from the ventral surface. This ventral wedge cut, which was tangent to midline, causes the brainstem block to sit slightly to the right when horizontal and orients the ST axons with the NTS in a common plane for cutting. The tissue block was then mounted horizontally to a pedestal and submerged in cold aCSF on a vibrating microtome (VT1000S; Leica Microsystems Inc., Bannockburn, IL). Approximately 300 μm was removed from the dorsal surface and then a single 250 μm thick horizontal slice was collected that contained the ST together with the neuronal cell bodies of the medial NTS region. Slices were cut with a sapphire knife (Delaware Diamond Knives, Wilmington, DE). Slices were submerged in aCSF and secured with a fine polyethylene mesh in a perfusion chamber with continuous perfusion of aCSF bubbled with 95% O_2_–5% CO_2_ at 32–35°C and 300 mOsm.

### Whole cell patch-clamp recordings

Recorded neurons were located medial to the ST and within 200 μm rostral or caudal of obex. Recording electrodes (2.8–3.8 MΩ) were filled with an intracellular solution containing (mM): 6 NaCl, 4 NaOH, 130 K-gluconate, 11 EGTA, 1 CaCl_2_, 1 MgCl_2_, 10 HEPES, 2 Na_2_ATP, and 0.2 Na_2_GTP. The intracellular solution was pH 7.4 and 296 mOsm. All neurons were studied under voltage clamp conditions with an Axopatch 200A or MultiClamp 700A amplifier (Molecular Devices, Union City, CA) and held at *V_H_* = −60 mV using pipettes in whole cell patch configuration. Signals were filtered at 3 kHz and sampled at 30 kHz using p-Clamp software (version 10, Molecular Devices). Liquid junction potentials were not corrected.

### Functional identification of second-order NTS neurons

In order to selectively activate ST afferent fibers a concentric bipolar stimulating electrode (200 μm outer tip diameter; Frederick Haer Co., Bowdoinham, ME) was placed on distal portions of the visible ST rostral to the recording region. Current shocks to the ST occurred every 6 s (shock duration 60 μs) using a Master-8 isolated stimulator (A.M.P.I., Jerusalem, Israel). Suprathreshold shocks resulted in long latency excitatory postsynaptic currents (EPSCs) resulting from the synchronous release of multiple glutamate vesicles activating postsynaptic α-amino-3-hydroxyl-5-methyl-4-isoxazole-propionate (AMPA) glutamate receptors under these recording conditions. Latency was measured as the time between the ST shock artifact and the onset of the resulting synchronous EPSC. Synaptic “jitter” was calculated as the standard deviation of ST-EPSC latencies for 30–40 trials within each neuron. Jitters of <200 μs functionally identify monosynaptic afferent inputs onto the recorded NTS neuron (Doyle and Andresen, 2001). The TRPV1 selective agonist capsaicin (CAP) was applied to identify ST afferents as possessing TRPV1 or not. Specifically, CAP exposure dramatically increases the frequency of sEPSCs and decreases the evoked synchronous ST-EPSCs in brainstem slices (Peters et al., 2011). CAP exposure on dissociated nodose neurons produces a large increase in cytosolic calcium (Figure 8).

### Statistical analysis of synaptic responses

Digitized waveforms of synaptic events were analyzed using an event detection and analysis program (MiniAnalysis, Synaptosoft, Decatur, GA) for all quantal synaptic currents and Clampfit 10 (Molecular Devices) for all ST-stimulated currents. All events >10 pA were counted for frequency values. Fitting of quantal EPSC amplitudes and decay kinetics (90–10%) was performed using a fitting protocol (MiniAnalysis) on >100 discrete events. To quantify the number of events released asynchronously following ST stimulation we summed the total number of quantal events for 4 s following the synchronous EPSCs from across 50 trials. In order to account for the ongoing spontaneous release we subtracted the number of quantal events for the same period of time during unstimulated conditions. For statistical comparisons *T*-tests, Mann Whitney Rank Sum test, and linear regression analysis were used when appropriate (Systat Software Inc., San Jose, CA). For group comparisons *p* < 0.05 was considered statistically significant.

### Nodose ganglia isolations and primary neuronal cultures

Nodose ganglia were isolated bilaterally from rats and mice under a deep plane of anesthesia (Ketamine, 25 mg/100 g; with Xylazine, 2.5 mg/100 g) using aseptic surgical conditions based on methods previously reported (Lancaster and Weinreich, 2001; Simasko et al., 2002). Following a midline incision in the neck the musculature was retracted and blunt dissection techniques were used to dissociate the vagal trunk from the common carotid artery. High-magnification optics (10–100x dissecting scope; Leica Microsystems, Buffalo Grove, IL) were utilized to help visualize the nodose ganglia and facilitate complete removal. Once isolated, nodose ganglia were digested in Ca^2+^/Mg^2+^free Hank's Balanced Salt Solution containing 1 mg/mL of both Dispase II (Hoffmann- La Roche) and Collagenase Type 1A (Sigma Aldrich) (120 min at 37°C in 95% air/5% CO_2_). Neurons were dispersed by gentle trituration through silicanized pipettes, and then washed in Dulbecco's Modified Eagle's Medium (DMEM) supplemented with 10% fetal bovine serum (FBS) and 1% penicillin-streptomycin. Dispersed cells were plated onto poly-lysine coated coverslips and maintained in DMEM+10% FBS (37°C in 95% air/5% CO_2_). Measurements were made within 24 h of isolation.

### Ratiometric fluorescent calcium measurements

Intracellular calcium measurements were made with the fluorescent Ca^2+^ indicator Fura-2 AM (Molecular Probes, Eugene, OR). Manipulations were made at room temperature in a physiological saline bath (in mM: 140 NaCl, 5 KCl, 2 CaCl_2_, 1 MgCl_2_, 6 glucose, 10 HEPES with pH adjusted to 7.4 with NaOH). High K^+^ bath (HiK) had 55 mM KCl with an equimolar reduction of NaCl to 90 mM. Neurons on coverslips were loaded with 1 μM Fura-2-AM for 1 h followed by a 15 min wash for de-esterification. Coverslips were mounted into an open chamber and constantly perfused with physiological bath. Neurons containing Fura-2 were alternatively excited with 340 and 380 nm light with fluorescence monitored at 510 nm. Data points were collected with MetaFluor software at 6 s time points. Ratios of fluorescence intensity were converted to Ca^2+^ concentrations with a standard curve.

### Statistical analysis for calcium imaging experiments

For each experiment data were collected from 2 to 3 nodose ganglion cell cultures. Protocols were designed to be within subject and analyzed using repeated measures ANOVA followed by *post-hoc* comparisons against control. Parameters of dose-response relationships (EC_50_, slope, maximum) were determined by sigmoid fit of the data. For antagonist studies (ruthenium red) all neurons received each treatment and were compared using within subject *t*-tests. Data are expressed as the average ± s.e.m. Statistical analysis was performed using SigmaStat software (Systat Software Inc., San Jose, CA).

## Results

**Figure 1:**
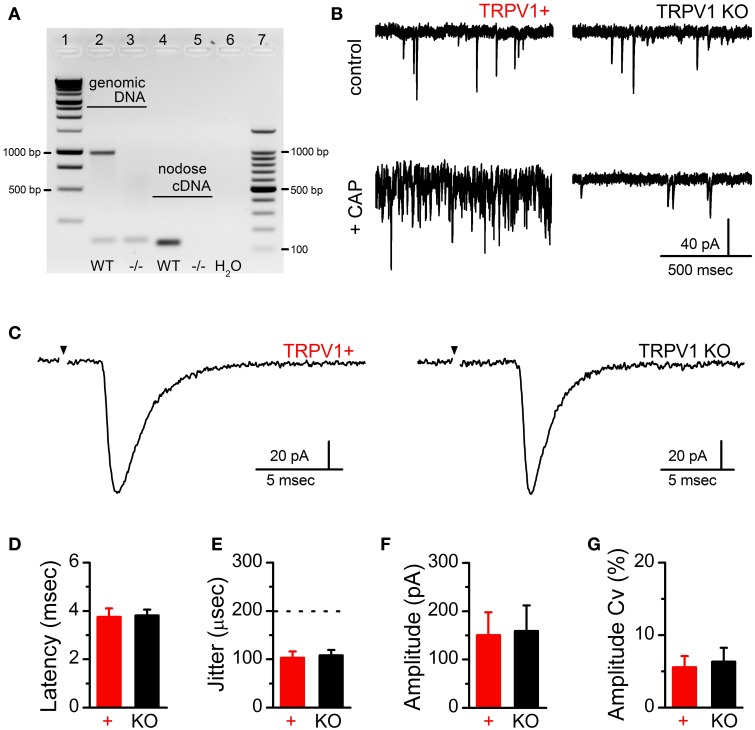
**Confirmation of TRPV1 deletion and comparison of ST-evoked synchronous glutamate release between primary afferent contacts onto second-order NTS neurons from control and TRPV1 KO animals**. **(A)** Gel electrophoresis showing the TRPV1 specific genomic DNA (predicted band at 984 bp) and nodose cDNA (predicted band at 134 bp) amplicon from control and TRPV1 KO mice. **(B)** Currents traces showing sEPSC frequency in control bath (upper panels) and following CAP (100 nM) exposure (lower panels) from TRPV1+ (left) and TRPV1 KO afferents. **(C)** Current traces comparing ST-evoked synchronous glutamate release from single ST afferent inputs confirmed to express TRPV1 (CAP sensitive) or from TRPV1 KO mice. Traces are of individual sweeps with the black arrow indicating the time of ST activation. The stimulus artifacts have been removed. There were no significant differences between control (*n* = 6) and TRPV1 KO (*n* = 13) ST afferent inputs with regards to the average latency to EPSC onset **(D)** or synaptic “jitter” (the dotted line indicates the threshold below which inputs are considered monosynaptic) **(E)** (*P* = 0.56 and *P* = 0.74 respectively, unpaired *t*-tests); demonstrating monosynaptic innervation from ST afferents likely expressing similar degrees of myelination. Further, neither the amplitude **(F)** nor coefficient of amplitude variation (Cv) **(G)** of single ST-EPSCs were significantly different (*P* = 0.93 and *P* = 0.95 respectively, unpaired *t*-tests); consistent with similar numbers of vesicle release sites (N) and release probability (P_*r*_).

Action potential invasion of vagal afferent terminals produces synchronous release of multiple glutamate vesicles via opening of N-type voltage activated calcium channels (Mendelowitz et al., 1995). While TRPV1 activation increases terminal calcium and vesicle mobilization, it is not reported to contribute to synchronous vesicle release (Peters et al., 2010). As such we predict that genetic deletion of TRPV1 will leave evoked synchronous ST EPSCs intact. To confirm that TRPV1 gene expression was eliminated, we probed the gene product from genomic DNA and RNA expression within the nodose ganglia (Figure 1A). We also functionally confirmed the loss of TRPV1 by CAP induced increases in frequency of spontaneous EPSCs which were absent in TRPV1^−/−^ synapses (Figure 1B). The horizontal brainstem slice permits selective activation of the ST bundle by placement of a concentric bipolar stimulating electrode distant (1–3 mm) from the recorded neurons (Doyle et al., 2004). Using graded stimulation intensities we are able to recruit single or multiple ST afferents discretely (McDougall et al., 2009; Peters et al., 2011). We found that the amplitude of solitary tract evoked EPSCs (ST-EPSCs) from single ST afferent contacts were not statistically different between control and TRPV1 KO animals (control: 150 ± 47 pA vs. TRPV1^−/−^: 158 ± 53 pA, *N* = 6 and 13 respectively, *P* = 0.93, *T*-test) (Figure 1). The latency to event onset is an approximate measure of conduction velocity and was also not different between groups (control: 3.58 ± 0.35 ms vs. TRPV1^−/−^: 3.82 ± 0.24 ms, *N* = 6 and 13 respectively, *P* = 0.56, *T*-test) (Figure 1B); indicating the afferent fibers activated have the same degree of myelination. The variability, or “jitter,” in latency is used as a verified functional determinate of connectivity with jitter measurements under 200 μs indicating direct monosynaptic ST afferent innervation of the recorded neurons (control: 101 ± 17 μs vs. TRPV1^−/−^: 108 ± 11 μ s, *N* = 6 and 13 respectively, *P* = 0.74, *T*-test) (Figure 1C).

**Figure 2:**
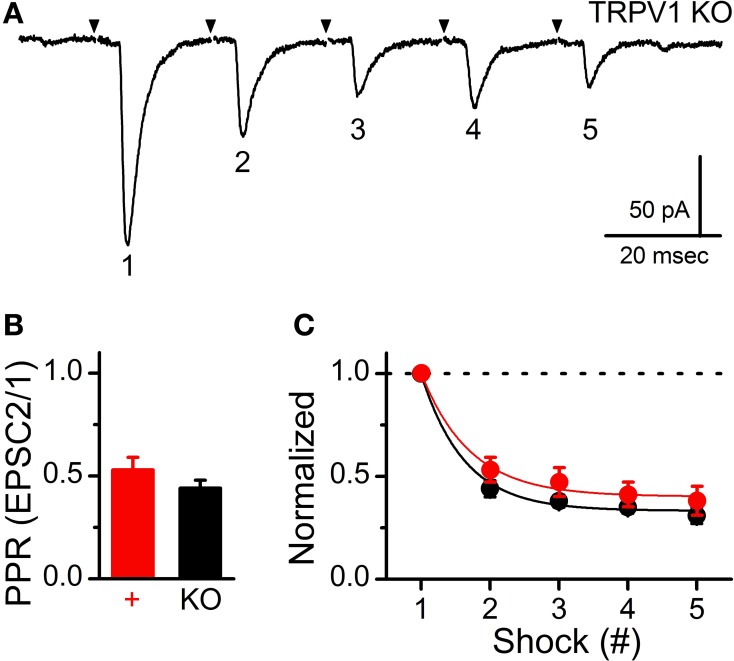
**Conserved synaptic performance during trains of ST shocks between control and TRPV1 KO afferents**. **(A)** Representative current trace showing decreasing ST-evoked EPSCs during a train of ST shocks (5 shocks at 50 Hz). Traces are of individual sweeps with the black arrow indicating the time of ST activation. The stimulus artifacts have been removed. This frequency dependent depression (FDD) is a result of a high initial probability of vesicle release (P_*r*_) and relatively fast stimulations releasing vesicles faster than they can be mobilized to the membrane. **(B)** The paired-pulse ratio (PPR) comparison between evoked EPSC2 to EPSC1 (20 ms interval) showed no significant difference between groups (*P* = 0.08, unpaired *t*-test); consistent with similar initial P_*r*_. **(C)** The train a stimulations produced nearly identical frequency dependent depression (FDD) profiles that were not different between groups (*P* = 0.93, ANOVA). Dashed line provides reference to initial EPSC1 amplitude.

On average, ST-EPSC amplitude and variability (amplitude co-variance, Cv) were also not different between control and TRPV1 KO afferents (control: 5.58 ± 1.54% vs. TRPV1^−/−^: 6.34 ± 1.92%, *N* = 6 and 13 respectively, *P* = 0.96, *T*-test) (Figure 1E); suggesting similar numbers of synaptic release sites (N) and probability of vesicle release (P_*r*_). To compare potential differences in synaptic performance between these two groups we delivered trains of ST stimulations (5 shocks at 50 Hz) and compared the initial paired-pulse ratio (PPR) and frequency dependent depression (FDD) (Figure 2). The PPR between control and TRPV1 KO was not different, consistent with similar initial P_*r*_ (control: 0.53 ± 0.06 vs. TRPV1^−/−^: 0.44 ± 0.04, *N* = 6 and 13 respectively, *P* = 0.08, *T*-test) (Figure 2B). Initial FDD reflects depletion of the readily releasable pool of vesicles followed by the steady-state equilibrium between vesicle mobilization and release (Figure 2C). Loss of TRPV1 did not significantly alter either of these parameters (*P* = 0.93, ANOVA).

**Figure 3:**
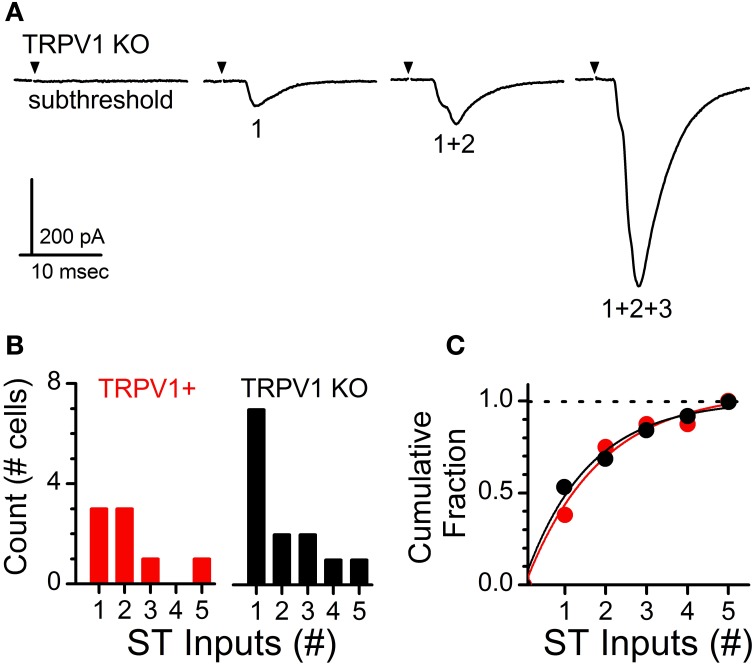
**Analysis of primary afferent innervation patterns onto second-order NTS neurons from control and TRPV1 KO animals**. **(A)** Current traces showing increasing stimulus intensity recruiting a single contact followed by multiple convergent ST afferent inputs. Traces are of individual sweeps with the black arrow indicating the time of ST activation. The stimulus artifacts have been removed. Activation of multiple inputs produces a compound waveform; with individual inputs discriminated by shock intensity at activation, latency, and amplitude. **(B)** Histogram detailing the total number direct ST inputs across recorded neurons from each group. **(C)** Cumulative proportion showing no significant difference in the percentage of neurons receiving 1 or >1 inputs between control and TRPV1 KO animals (*P* = 0.63, Wilcoxon ranked sum test). Dashed line provides reference to 1.0 on the vertical axis.

Increasing shock intensity on the ST bundle routinely activates multiple direct afferent inputs. Convergent inputs are discriminated based on threshold of activation for each recruited ST-EPSC, latency to ST-EPSC onset, and cumulative amplitude of the multiple ST-EPSCs (i.e., max current change from 1 input alone, 1 + 2 inputs, 1 + 2 + 3 inputs, etc.) (Figure 3A) (McDougall et al., 2009; Peters et al., 2011). Using the systematic stimulus recruitment analysis a functional map of the number of direct ST afferent inputs innervating each medial NTS neuron was generated and compared between control and TRPV1 KO recordings (Figures 3B,C). Comparison of innervation patterns across neurons revealed no significant difference in the ST afferent innervation patterns between control and TRPV1 KO animals (*P* = 0.63, Wilcoxon ranked sum test).

**Figure 4:**
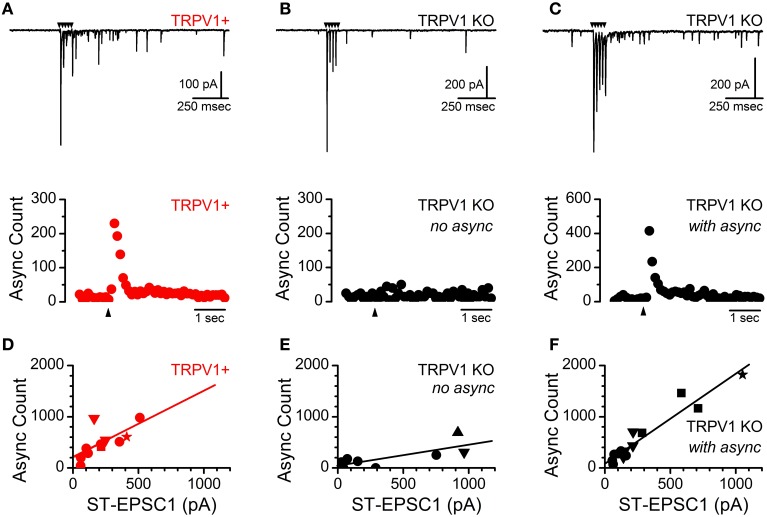
**Identification of a subpopulation of ST afferents from TRPV1 KO mice which maintain activity dependent asynchronous glutamate release**. (**A**, upper) Current trace showing ST driven synchronous and asynchronous release from an identified TRPV1+ afferents in control mice. (**A**, lower) Histogram of summed asynchronous quantal glutamate events from across 50 trials. Black arrows indicate time of ST shock in all panels. (**B**, upper/lower) Nearly half (6 of 13) of all neurons recorded from TRPV1 KO ST afferents showed no asynchronous release regardless of the size or number of direct ST inputs. **(C**, upper**)** A significant population of TRPV1 KO afferents still mobilized vesicles via the asynchronous release pathway; with the magnitude and duration of additional release similar to the TRPV1+ afferents from control animals **(C**, lower**)**. **(D)** The total number of asynchronously released vesicles positively correlates with the size of the evoked ST-EPSC (*n* = 11 inputs, *R*^2^ = 0.48, slope = 1.34 ± 0.47) from control TRPV1+ afferents. **(E)** TRPV1 KO afferents with no asynchronous release show only a weak relationship between EPSC1 amplitude and additional quantal events (*n* = 8 inputs, *R*^2^ = 0.58, slope = 0.42 ± 0.14). **(F)** From TRPV1 KO afferents with asynchronous release the magnitude of the release was strongly correlated with the size of the evoked synchronous EPSC (*n* = 15 inputs, *R*^2^ = 0.92, slope = 1.75 ± 0.14). The synchronous to asynchronous relationship is maintained across multiple inputs and the data have been fit including convergent ST contacts: 1st = circle, 2nd = down triangle, 3rd = square, 4th = star, and 5th = upward triangle. These data suggest an additional mechanism of mobilizing asynchronous glutamate vesicles is present in a subpopulation of ST afferents.

Action-potential invasion of the central terminals of ST afferents produces rapid synchronous vesicle mobilization followed by prolonged TRPV1 dependent asynchronous vesicle release in rats (Peters et al., 2010). Both of these release pathways are also present in ST afferents from mice however the asynchronous profile in mice decays with a single exponential time constant as compared to a biexponential decay in the rat and as a result the synchronous to asynchronous ratio is increased. Nevertheless, activation of TRPV1+ ST afferents in mice produces a reliable asynchronous release that peaks rapidly and decays over a period of hundreds of milliseconds and is proportional to the size of the evoked ST-EPSC (*n* = 11 inputs, *R*^2^ = 0.48, slope = 1.34 ± 0.47 sync/async ratio) (Figures 4A, upper/lower panels, and D). As in the rat, convergent ST inputs segregate based on the presence or absence of TRPV1 and asynchronous release pathway. As a result all convergent inputs onto a second-order NTS neuron either possess asynchronous release or not (Figure 4D). In TRPV1 KO animals nearly half of recorded neurons (6 of 13) received direct ST afferent innervation with no asynchronous release (*n* = 8 inputs, *R*^2^ = 0.58, slope = 0.42 ± 0.14 sync/async ratio) (Figures 4B, upper/lower panels, and E) while the remaining neurons received afferent innervation with asynchronous release indistinguishable from the control animals (*n* = 15 inputs, *R*^2^ = 0.92, slope = 1.75 ± 0.14 sync/async ratio) (Figures 4C, upper/lower panels, and F). Further, recruitment of multiple direct inputs either completely lacked or possessed asynchronous release (Figures 4E,F). These results were surprising for several reasons; first we would have predicted that asynchronous release would have been attenuated and not completely lacking or completely present; and second, ST afferent innervation is still segregated based off the presence or absence of asynchronous release.

**Figure 5:**
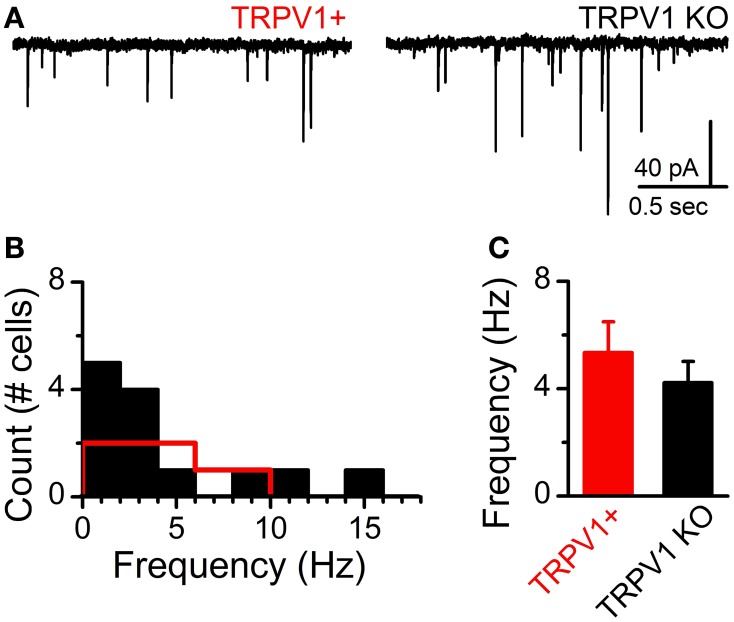
**Comparison of sEPSCs frequency onto NTS neurons from control (TRPV1+) and TRPV1 KO mice**. **(A)** Current traces (*V*_m_ = −60 mV) of sEPSCs onto NTS neurons with each type of identified direct ST afferent contact. Temperature was held constant at 33°F. **(B)** Histogram of average sEPSC frequency across neurons (TRPV1+: *n* = 8 neurons, red and TRPV1 KO: *n* = 13 neurons, black). **(C)** The distribution of sEPSC frequencies was not statistically different between neurons receiving TRPV1+ and TRPV1 KO contacts (*P* = 0.57, unpaired *t*-test).

**Figure 6:**
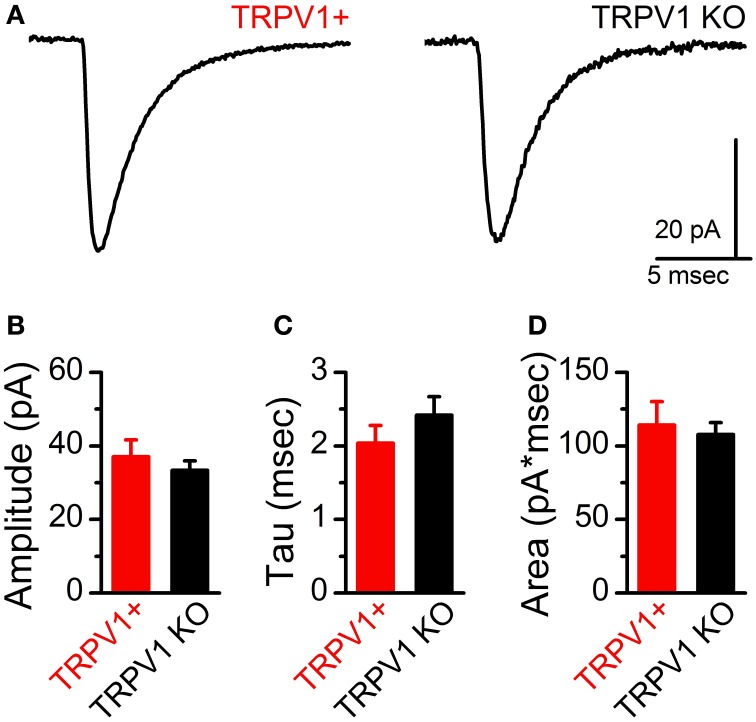
**No difference in spontaneous EPSC waveforms between control and TRPV KO ST afferents. (A)** Representative average sEPSCs from control and TRPV1 KO afferents. Waveforms are the average of 100 events. Statistical comparison of sEPSC amplitude **(B)** and decay kinetics **(**Tau, **C)** and integrated current **(**Area, **D)** were not significantly different between TRPV1+ and TRPV1 KO afferents (*P* = 0.46, 0.71, and 0.31 respectively, unpaired *t*-tests).

**Figure 7:**
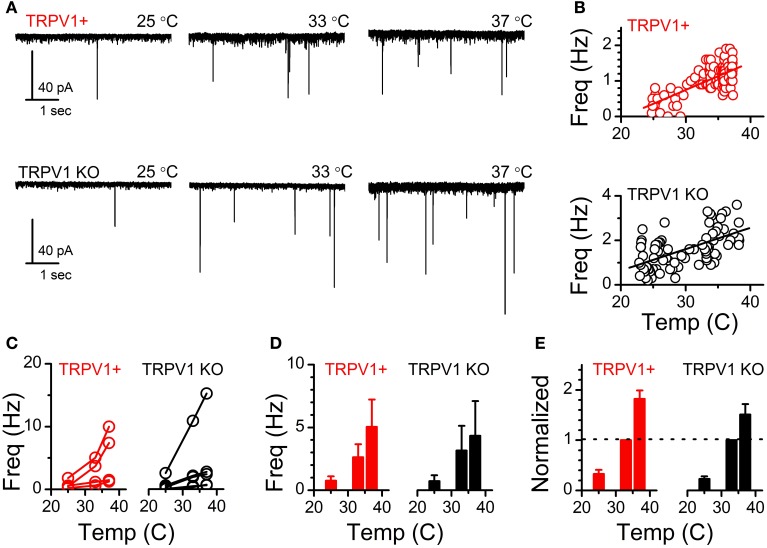
**Temperature driven changes in sEPSC frequency across the physiological range are not different from TRPV1 KO afferents**. **(A)** Current traces of temperature driven increase in sEPSC frequency from TRPV1+ and TRPV1 KO mice. **(B)** Scatter-plot showing binned average frequency (10 s bins) across changing temperatures from the TRPV1+ and TRPV1 KO neurons shown in **(A)**. **(C)** Frequency of sEPSCs at each temperature from individual recorded neurons from TRPV1+ and TRPV1 KO animals. Lines connect frequencies at each temperature for an individual neuron. **(D)** Average sEPSC frequencies across neurons at each of the steady state temperatures. **(E)** Changes in frequency normalized to frequency recorded at 33°C. The frequency of spontaneous release is still temperature driven in the absences of TRPV1.

At ST-NTS synapses ongoing spontaneous vesicle fusion and release is steeply temperature dependent and suspected to be directly controlled by TRPV1 (Shoudai et al., 2010). As such we may expect differences in spontaneous release between control and TRPV1 KO animals. Initially, we compared the steady-state frequency of sEPSCs from control and TRPV1 KO animals at a constant warm recording temperature (33°C) and found no significant difference (control: 4.43 ± 1.04 Hz vs. TRPV1^−/−^: 4.17 ± 1.17 Hz, *N* = 8 and 13 respectively, *P* = 0.57, unpaired *T*-test) (Figure 5). While there exists a wide range of sEPSC frequencies neither the distributions nor the average frequencies were statistically different between groups (Figures 5B,C). This observation suggests that at warm temperatures at least the rate of spontaneous vesicle release is maintained independent of TRPV1. Detailed fitting analysis of the quantal EPSCs also found no differences in event amplitude(control: 37 ± 5 pA vs. TRPV1^−/−^: 32 ± 3 pA, *P* = 0.59, *T*-test), decay (tau) (control: 2.04 ± 0.24 ms vs. TRPV1^−/−^: 2.52 ± 0.27 ms, *P* = 0.17, *T*-test), or total charge transfer (control: 114 ± 16 pA^*^ms vs. TRPV1^−/−^: 105 ± 7 pA^*^ms, *P* = 0.98, *T*-test) (Figure 6). These findings are consistent with similar vesicle neurotransmitter content, postsynaptic receptor expression and position on the recorded neurons (distal vs. proximal to the soma). Varying bath temperature from 25 to 39°C revealed that frequency of spontaneous release was still temperature dependent from controls (*N* = 4, *P* = 0.005, Friedman repeated measures ANOVA) and TRPV1^−/−^ (*N* = 5, *P* < 0.001, Friedman repeated measure ANOVA) animals; but not statistically different between control and TRPV1^−/−^ animals (*P* = 0.82, Two-Way ANOVA) (Figure 7). Together these findings indicate that TRPV1 may be involved in driving spontaneous fusion at very high temperatures (>40°C) while additional temperature sensitive mechanisms appear to be maintaining release at lower, physiological temperatures.

**Figure 8:**
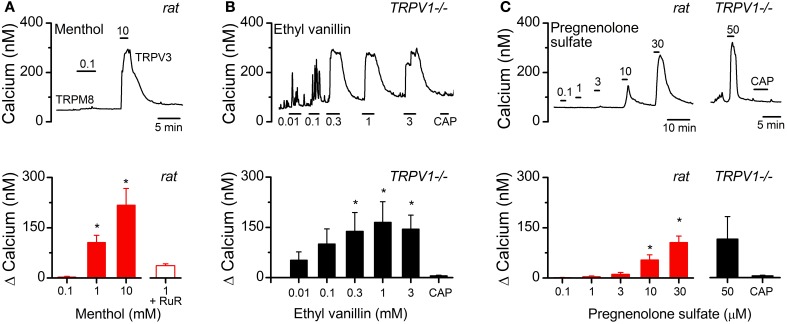
**Functional evidence suggesting alternative thermo-TRP channels present in vagal afferents**. **(A**, upper panel**)** Representative calcium trace from cultured vagal afferent cell bodies showing menthol fails to activate at concentrations selective for TRPM8, but produce large calcium transients at concentrations known to activate TRPV3. **(A**, lower panel**)** Average concentration response relationship for menthol induced increases in cytosolic calcium (*N* = 7, *P* < 0.001, ANOVA). Menthol induced calcium increases were significantly reduced with RuR (*N* = 9, *P* < 0.001, paired *t*-test) also consistent with TRPV3 activation. **(B**, upper panel**)** Calcium trace showing the concentration-dependent increase in cytosolic calcium by the TRPV3 agonist ethyl vanillin in cultured vagal afferents taken from TRPV1^−/−^ mice. CAP exposure failed to elicit a response as predicted. **(B**, lower panel**)** Average concentration response relationship for ethyl vanillin induced increases in cytosolic calcium (*N* = 5, *P* = 0.002, ANOVA). **(C**, upper panel**)** Left: Calcium trace showing the TRPM3 agonist pregnenolone sulfate concentration-dependently activated a subpopulation of CAP-sensitive vagal afferent neurons. Right: This response was maintained in afferents taken from TRPV1^−/−^ mice. **(C**, lower panel**)** Average change in intracellular calcium across concentrations from pregnenolone sulfate responsive neurons from rat (*N* = 8, *P* < 0.001, ANOVA) and TRPV1^−/−^ mice (*N* = 3, *P* < 0.01, paired *T*-test). Statistical significance is indicated by an asterisk (*).

The findings so far suggest a more nuanced contribution of TRPV1 to synaptic glutamate release and indicate the presence of additional temperature dependent controls of quantal vesicle release. Because spontaneous release was maintained across warm temperatures and is known to be calcium dependent (Smith et al., 2012) we hypothesized that additional thermosensitive TRP channels are expressed in vagal primary afferents. Only a few thermosensitive TRP channels have temperature activation thresholds in the warm range including TRPV3 and TRPM3 (Ramsey et al., 2006). To begin to determine if these channels are present and functional we utilized fluorescent calcium imaging of cultured nodose ganglion neurons from control rats and TRPV1^−/−^ mice (Figure 8). TRPV3 has no described selective agonist but can be activated by the TRPM8 agonist menthol at high concentrations (*N* = 7, *P* < 0.001, Kruskal-Wallis One-Way ANOVA) (Macpherson et al., 2006). TRPV3 mediated responses were differentiated from TRPM8 based on threshold to activation and blockade by bath application of ruthenium red (menthol 1 mM: 106 ± 21 nM vs. menthol + RuR: 37 ± 5 nM, *N* = 9, *P* = 0.006, paired *T*-Test) (Figure 8A, upper and lower panels). We also screened the TRPV3 agonist ethyl vanillin on cultured vagal afferents from TRPV1^−/−^ mice and found that it concentration-dependently increased cytosolic calcium in a subpopulation of neurons (ethyl vanillin 0.01, 0.1, 0.3, 1, 3 mM: *N* = 5, *P* = 0.002, One-Way repeated measure ANOVA) (Figure 8B, upper and lower panels); suggesting TRPV3 expression is maintained independent of TRPV1. The ion channel TRPM3, on the other hand, is selectively activated by the steroid pregnenolone sulfate (pregnenolone sulfate 0.1, 1, 3, 1, 10, 30 μM: 1 ± 1, 4 ± 2, 11 ± 6, 54 ± 15, 106 ± 19 nM respectively, *N* = 8, *P* < 0.001, One-Way repeated measure ANOVA) (Figure 8C) (Wagner et al., 2008). Pregnenolone sulfate activated a subpopulation of cultured afferents from rats and TRPV1^−/−^ mice (115 ± 67, *N* = 3, *P* = 0.11) (Figure 8C). In both experiments responsive neurons were also activated by the TRPV1 agonist capsaicin (data not shown) showing co-segregation of TRPV3 and TRPM3 with TRPV1. CAP exposure in the TRPV1^−/−^ neurons failed to increase cytosolic calcium confirming deletion of the TPRV1 gene.

## Discussion

The major findings of these experiments confirm that TRPV1 is important in asynchronous vesicle release pathways at ST-NTS synapses but suggest that additional thermosensitive TRP channels are present in the presynaptic primary afferent neurons and may be contributing to the remaining asynchronous release and temperature driven spontaneous glutamate release. The fact that convergent primary afferent innervation remains completely segregated based on the presence or absence of asynchronous vesicle release, independent of TRPV1 expression, indicates a particular importance for asynchronous neurotransmission in ST-NTS synaptic organization. The maintenance of overall synaptic innervation patterns and action-potential driven synchronous vesicle release from TRPV1 KO afferents suggest that TRPV1 signaling and its impact on quantal glutamate release are not essential for development of this circuitry. Rather, TRPV1, likely in concert with other thermosensitive TRP channels, provides precise and nuanced control mechanism of quantal glutamate release pathways.

### Action-potential evoked synchronous release is conserved in TRPV1 KO afferents

Neither primary afferent innervation patterns, action-potential driven synchronous release, nor vesicle mobilization to the readily releasable pool during trains of stimulations were different in TRPV1 KO afferents compared to control animals. Considering TRPV1 is expressed in the majority (70–80%) of visceral afferent neurons forming synapses onto second-order NTS neurons (Andresen et al., 2012b) and provides a large calcium influx pathway, we were surprised that none of these parameters were altered. In particular during trains of action-potentials, where terminal calcium waves accumulate and TRPV1 is likely being activated by voltage and other factors such as anandamide generation and activation of protein kinase C (PKC) (Premkumar and Ahern, 2000; Di Marzo et al., 2001; Studer and McNaughton, 2010), showed no difference in the vesicle mobilization. Other use dependent calcium influx pathways, such as activation of presynaptic NMDA type glutamate receptors are known to facilitate synapsin phosphorylation (Campos et al., 2013); a key step in vesicle shuttling to the readily releasable pool (Cesca et al., 2010). The current findings are consistent with the concept that calcium influx into the presynaptic terminal is very precisely controlled and that different calcium influx pathways participate in distinct cellular processes (Kavalali et al., 2011). It appears that TRPV1 derived calcium has little to do with synchronous release pathways and is specialized to impact quantal vesicle mobilization via spontaneous and asynchronous release pathways (Andresen et al., 2012a).

### The complete presence or absence of asynchronous release from TRPV1 KO afferents

The presence of TRPV1 predicts robust action-potential driven asynchronous release (Peters et al., 2010). Pharmacological blockade of TRPV1 attenuates the asynchronous release pathway (Peters et al., 2010) and suggests that TRPV1 genetic deletion should diminish asynchronous release for a given synapse. Surprisingly, the present work comparing ST-NTS synapses between control and TRPV1 KO animals revealed that afferents genetically lacking TRPV1 either completely lacked asynchronous release or completely maintained it (Figure 4). The characterization of the inputs lacking asynchronous release suggest that they are not simply an improbable collection of neurons that happen to have all A-fiber afferent inputs, based on their conduction velocity (latency), size of quantal events, and distribution within the medial NTS (Peters et al., 2010, 2011). Rather, they may represent the afferents wherein the asynchronous release would have been maintained entirely by the presence of TRPV1 and those with the remaining asynchronous release have redundant use dependent calcium influx pathways, such as the alternative TRP channels (TRPV3 and TRPM3) characterized in Figure 8. Convergent ST afferent innervation is segregated based on the presence or absence of TRPV1 and asynchronous release in control animals (Peters et al., 2011). Surprisingly TRPV1 KO afferents maintained this relationship. Wherein ST afferents without asynchronous release converged onto common neurons, those with asynchronous release converged onto a separate population of NTS neurons. This observation suggests that the presence or absence of the asynchronous vesicle release pathway may determine of convergent afferent organization into the NTS.

### Temperature driven spontaneous glutamate release and the presence of alternative thermosensitive TRP channels

Spontaneous vesicle release is a stochastic process largely driven by temperature sensitive calcium conductances at ST-NTS synapses (Shoudai et al., 2010). The presence of TRPV1 in ST primary afferents predicts relatively high spontaneous frequency; while pharmacological antagonism of TRPV1 reduces overall temperature sensitivity of the spontaneous release pathway particularly at the highest temperatures recorded (~40°C) (Peters et al., 2010; Shoudai et al., 2010). In TRPV1 KO animals we found that frequency of spontaneous release was indistinguishable from control animals (Figure 5). Further, temperature ramps revealed statistically significant increases in spontaneous frequency from 33 to 37°C that was not different between control and TRPV1^−/−^ animals (Figure 7). The observation that spontaneous frequency is maintained at warm temperatures and still reactive to temperature steps from 25 to 37°C indicates that additional temperature sensitive mechanisms are intact in the TRPV1 KO afferents. These temperature driven changes may be relevant in detecting a number of physiological processes that alter internal temperatures such as post prandial thermogenesis (Griggio et al., 1991), circadian temperature changes (Buhr et al., 2010), or infection. Our preliminary pharmacological findings demonstrating functional expression of TRPV3 and TRPM3 provide evidence for plausible candidate ion channels to maintain the temperature driven spontaneous release at warm physiological temperatures. However, the specific roles of these candidate channels in the control of synaptic glutamate release remains to be determined.

Together these findings suggest additional temperature dependent mechanisms controlling asynchronous and thermosensitive spontaneous release at physiological temperatures, possibly mediated by additional thermosensitive TRP channels in primary afferent terminals. Because a major portion of the synaptic charge transfer is carried by glutamate released via spontaneous and asynchronous release pathways these central thermo-sensitive TRP channels are likely critical in the control of autonomic reflex generation and neuronal processing originating via cranial visceral afferents.

## Grants

Supported by a grant from the National Institutes of Health, DK092651.

### Conflict of interest statement

The authors declare that the research was conducted in the absence of any commercial or financial relationships that could be construed as a potential conflict of interest.

## References

[B1] Andresen M. C. Fawley J. A. Hofmann M. E. (2012a). Peptide and lipid modulation of glutamatergic afferent synaptic transmission in the solitary tract nucleus. Front. Neurosci. 6:191 10.3389/fnins.2012.00191 23335875 PMC3541483

[B2] Andresen M. C. Hofmann M. E. Fawley J. A. (2012b). The unsilent majority-TRPV1 drives “spontaneous” transmission of unmyelinated primary afferents within cardiorespiratory NTS. Am. J. Physiol. Regul. Integr. Comp. Physiol. 303, R1207–R1216 10.1152/ajpregu.00398.2012 23076872 PMC3532589

[B3] Bailey T. W. Hermes S. M. Andresen M. C. Aicher S. A. (2006a). Cranial visceral afferent pathways through the nucleus of the solitary tract to caudal ventrolateral medulla or paraventricular hypothalamus: target-specific synaptic reliability and convergence patterns. J. Neurosci. 26, 11893–11902 10.1523/JNEUROSCI.2044-06.2006 17108163 PMC6674856

[B4] Bailey T. W. Jin Y.-H. Doyle M. W. Smith S. M. Andresen M. C. (2006b). Vasopressin inhibits glutamate release via two distinct modes in the brainstem. J. Neurosci. 26, 6131–6142 10.1523/JNEUROSCI.5176-05.2006 16763021 PMC2680488

[B5] Buhr E. D. Yoo S. H. Takahashi J. S. (2010). Temperature as a universal resetting cue for mammalian circadian oscillators. Science 330, 379–385 10.1126/science.1195262 20947768 PMC3625727

[B6] Campos C. A. Shiina H. Silvas M. Page S. Ritter R. C. (2013). Vagal afferent NMDA receptors modulate CCK-induced reduction of food intake through synapsin I phosphorylation in adult male rats. Endocrinology 154, 2613–2625 10.1210/en.2013-1062 23715865 PMC3713210

[B7] Cesca F. Baldelli P. Valtorta F. Benfenati F. (2010). The synapsins: key actors of synapse function and plasticity. Prog. Neurobiol. 91, 313–348 10.1016/j.pneurobio.2010.04.006 20438797

[B8] Chen C. Y. Horowitz J. M. Bonham A. C. (1999). A presynaptic mechanism contributes to depression of autonomic signal transmission in NTS. Am. J. Physiol. 277, H1350–H1360 10516169 10.1152/ajpheart.1999.277.4.H1350

[B9] Di Marzo V. Bisogno T. De Petrocellis L. (2001). Anandamide: some like it hot. Trends Pharmacol. Sci. 22, 346–349 10.1016/S0165-6147(00)01712-0 11431028

[B10] Doyle M. W. Andresen M. C. (2001). Reliability of monosynaptic transmission in brain stem neurons *in vitro*. J. Neurophysiol. 85, 2213–2223 10.1113/jphysiol.2010.190611 11353036

[B11] Doyle M. W. Bailey T. W. Jin Y.-H. Appleyard S. M. Low M. J. Andresen M. C. (2004). Strategies for cellular identification in nucleus tractus solitarius slices. J. Neurosci. Methods 37, 37–48 10.1016/j.jneumeth.2004.02.007 15196825

[B12] Griggio M. A. Richard D. Leblanc J. (1991). The involvement of the sympathetic nervous system in meal-induced thermogenesis in mice. Int. J. Obes. 15, 711–715 1778656

[B13] Kavalali E. T. Chung C. Khvotchev M. Leitz J. Nosyreva E. Raingo J. (2011). Spontaneous neurotransmission: an independent pathway for neuronal signaling? Physiology (Bethesda) 26, 45–53 10.1152/physiol.00040.2010 21357902

[B14] Lancaster E. Weinreich D. (2001). Sodium currents in vagotomized primary afferent neurones of the rat. J. Physiol. 536, 445–458 10.1111/j.1469-7793.2001.0445c.xd 11600680 PMC2278870

[B15] Loewy A. D. (1990). Central autonomic pathways, in Central Regulation of Autonomic Functions, eds Loewy A. D. Spyer K. M. (New York, NY: Oxford), 88–103

[B16] Macpherson L. J. Hwang S. W. Miyamoto T. Dubin A. E. Patapoutian A. Story G. M. (2006). More than cool: promiscuous relationships of menthol and other sensory compounds. Mol. Cell. Neurosci. 32, 335–343 10.1016/j.mcn.2006.05.005 16829128

[B17] McDougall S. J. Peters J. H. Andresen M. C. (2009). Convergence of cranial visceral afferents within the solitary tract nucleus. J. Neurosci. 29, 12886–12895 10.1523/JNEUROSCI.3491-09.2009 19828803 PMC2797442

[B18] Mendelowitz D. Yang M. Reynolds P. J. Andresen M. C. (1995). Heterogeneous functional expression of calcium channels at sensory and synaptic regions in nodose neurons. J. Neurophysiol. 73, 872–875 7760142 10.1152/jn.1995.73.2.872

[B19] Peters J. H. McDougall S. J. Fawley J. A. Andresen M. C. (2011). TRPV1 marks synaptic segregation of multiple convergent afferents at the rat medial solitary tract nucleus. PLoS ONE 6:e25015 10.1371/journal.pone.0025015 21949835 PMC3176783

[B20] Peters J. H. McDougall S. J. Fawley J. A. Smith S. M. Andresen M. C. (2010). Primary afferent activation of thermosensitive TRPV1 triggers asynchronous glutamate release at central neurons. Neuron 65, 657–669 10.1016/j.neuron.2010.02.017 20223201 PMC2837850

[B21] Peters J. H. McDougall S. J. Kellett D. O. Jordan D. Llewellyn-Smith I. J. Andresen M. C. (2008). Oxytocin enhances cranial visceral afferent synaptic transmission to the solitary tract nucleus. J. Neurosci. 28, 11731–11740 10.1523/JNEUROSCI.3419-08.2008 18987209 PMC2585803

[B22] Premkumar L. S. Ahern G. P. (2000). Induction of vanilloid receptor channel activity by protein kinase C. Nature 408, 985–990 10.1038/35050121 11140687

[B23] Ramsey I. S. Delling M. Clapham D. E. (2006). An introduction to trp channels. Annu. Rev. Physiol. 68, 619–647 10.1146/annurev.physiol.68.040204.100431 16460286

[B24] Saper C. B. (2002). The central autonomic nervous system: conscious visceral perception and autonomic pattern generation. Annu. Rev. Neurosci. 25, 433–469 10.1146/annurev.neuro.25.032502.111311 12052916

[B25] Shoudai K. Peters J. H. McDougall S. J. Fawley J. A. Andresen M. C. (2010). Thermally active TRPV1 tonically drives central spontaneous glutamate release. J. Neurosci. 30, 14470–14475 10.1523/JNEUROSCI.2557-10.2010 20980604 PMC2976575

[B26] Simasko S. M. Wiens J. Karpiel A. Covasa M. Ritter R. C. (2002). Cholecystokinin increases cytosolic calcium in a subpopulation of cultured vagal afferent neurons. Am. J. Physiol. Regul. Integr. Comp. Physiol. 283, R1303–R1313 10.1152/ajpregu.00050.2002 12388458

[B27] Smith S. M. Chen W. Vyleta N. P. Williams C. Lee C. H. Phillips C. (2012). Calcium regulation of spontaneous and asynchronous neurotransmitter release. Cell Calcium 52, 226–233 10.1016/j.ceca.2012.06.001 22748761 PMC3433637

[B28] Studer M. McNaughton P. A. (2010). Modulation of single-channel properties of TRPV1 by phosphorylation. J. Physiol. 588, 3743–3756 10.1113/jphysiol.2010.190611 20693293 PMC2998224

[B29] Wagner T. F. Loch S. Lambert S. Straub I. Mannebach S. Mathar I. (2008). Transient receptor potential M3 channels are ionotropic steroid receptors in pancreatic beta cells. Nat. Cell Biol. 10, 1421–1430 10.1038/ncb1801 18978782

